# FBXL10 regulates cardiac dysfunction in diabetic cardiomyopathy via the PKC β2 pathway

**DOI:** 10.1111/jcmm.14146

**Published:** 2019-01-31

**Authors:** Leilei Yin, Yingying Fang, Tao Song, Dan Lv, Zheng Wang, Li Zhu, Zihui Zhao, Xinhua Yin

**Affiliations:** ^1^ Department of Cardiology the First Affiliated Hospital of Harbin Medical University Harbin China; ^2^ Department of Digestive Heilongjiang Institute of traditional Chinese Medicine Harbin China

**Keywords:** Apoptosis, Diabetic cardiomyopathy, FBXL10, Inflammation, oxidative stress, PKC β2

## Abstract

Diabetic cardiomyopathy (DCM) is a condition associated with significant structural changes including cardiac tissue necrosis, localized fibrosis, and cardiomyocyte hypertrophy. This study sought to assess whether and how FBXL10 can attenuate DCM using a rat streptozotocin (STZ)‐induced DCM model system. In the current study, we found that FBXL10 expression was significantly decreased in diabetic rat hearts. FBXL10 protected cells from high glucose (HG)‐induced inflammation, oxidative stress, and apoptosis in vitro. In addition, FBXL10 significantly activated PKC β2 signaling pathway in H9c2 cells and rat model. The cardiomyocyte‐specific overexpression of FBXL10 at 12 weeks after the initial STZ administration attenuated oxidative stress and inflammation, thereby reducing cardiomyocyte death and preserving cardiac function in these animals. Moreover, FBXL10 protected against DCM via activation of the PKC β2 pathway. In conclusion, FBXL has the therapeutic potential for the treatment of DCM.

## 1 INTRODUCTION

Diabetic cardiomyopathy (DCM) presents with structural and functional deficits in cardiac tissue in individuals with diabetes that are not associated with hypertension or other coronary artery disease.^1^, ^2^ DCM is a major risk factor for heart failure in individuals with diabetes, and at the cellular level, this condition is associated with significant cardiomyocyte depletion, alterations in extracellular matrix composition, and abundant tissue fibrosis.^3^, ^4^ Based on a previous study, diabetic males and females are at a two‐ and five‐fold increased risk of heart failure, respectively.^5^ Similarly, nearly half of diabetic individuals without coronary artery disease still exhibit diastolic dysfunction consistent with cardiac abnormalities.^6^ Blood glucose control is not sufficient to reduce pathological cardiomyopathy in these diabetic individuals, making the identification of any molecular mechanisms that can inhibit DCM progression vital to patient longevity.^7^, ^8^

Numerous factors ultimately work in concert to drive the development and progression of DCM, with oxidative stress, inflammation, and cellular apoptosis being key proximal drivers of this disease.^9^, ^10^, ^11^ The production of reactive oxygen species (ROS) in response to hyperglycemia or inflammation can adversely affect myocyte function, eventually resulting in myocyte death and ultimately in cardiac dysfunction.^12^ Myocyte death and ROS overproduction are both associated with interstitial fibrosis, thereby promoting yet another deleterious cardiac phenotype.^13^ Suppressing ROS production and concurrent inflammation and myocyte death is thus a clear potential avenue for combatting DCM in at‐risk individuals.^14^

FBXL10 is a histone demethylase and a component of the noncanonical polycomb repressive complex 1 (PRC1).^15^ This protein contains a CxxC zinc finger domain that can identify unmethylated CpG islands, as well as PHD, F‐box, and leucine‐rich repeat (LRR) domains that are important for PRC1 incorporation.^16^, ^17^ FBXL10 is important in the context of cancers, driving tumorigenesis, and promoting the survival and expansion of so‐called cancer stem cells in many distinct malignancies,^18^, ^19^ but whether FBXL10 has any role in cardiomyocytes remains unknown.

The protein kinase C (PKC) family consists of many distinct proteins with different functions and tissue distributions.^20^, ^21^ In cardiac tissue, PKC α and PKC β2 are the predominant PKC isoforms.^22^ Altered PKC expression and function has been linked to many cardiovascular disease‐associated issues including heart failure and ischemia, and PKC inhibition has been shown to protect against structural and functional issues.^23^ Hyperglycemia has been identified as a potential causative agent of PKC α and PKC β2 overexpression early in the development of DCM, potentially driving the disease‐associated pathology of DCM.^24^ However, how PKC β2 contributes precisely to this process at the molecular level remains unknown.

In the current study, our aim was to determine whether FBXL10 could protect diabetic rats from streptozocin (STZ)‐induced cardiac damage. Furthermore, the role of PKC β2 signaling pathway in the regulation of the protection of FBXL10 in STZ‐induced cardiac damage was investigated.

## 2 MATERIALS AND METHODS

### 2.1 Animals and animal models

All animal studies were conducted in a manner consistent with NIH guidelines and those of our hospital. Standard housing was used to maintain 6 to 8‐week‐old male Sprague‐Dawley rats. After a 12‐hour fast, the rats were intraperitoneally injected once with STZ (65 mg/kg). The presence of diabetes was determined by identifying rats that had fasting blood glucose levels >13.9 mmol/L upon three independent measurements. Twelve weeks after diabetes diagnosis, a single tail‐vein injection of adeno‐associated virus (AAV)‐FBXL10 (Ad‐FBXL10) or AAV‐GFP was given to the rats at 1 × 10^11^ particles/rat. Diabetic rats were grouped by a random number table.

### 2.2 Echocardiography and measurement of hemodynamic variables

The echocardiographic examination was conducted in accordance with previous protocols.^25^ Hemodynamic measurements were conducted with a microtip transducer catheter and the Millar Pressure‐Volume System. Both measurements were performed at 16 weeks after the final STZ injection, and then the animals were sacrificed.

### 2.3 Adenoviral vector construction

The pAdEasy Adenoviral Vector System^25^, ^26^ (Qbiogene, CA, USA) was used to prepare recombinant adenoviruses that expressed either FBXL10 (Ad‐FBXL10) or Ad‐LacZ (Vigene Bioscience, Jinan, China) according to standard protocols. Briefly, the FBXL10 or LacZ sequences were cloned into pShuttle‐CMV, followed by homologous recombination in BJ5183 bacteria. The recombinant plasmids were maintained in parallel in HEK 293 cells.

### 2.4 Cell culture and stimulation

Neonatal rat cardiomyocytes were collected as previously described.^27^ H9c2 cells were purchased from Sigma. To assess the protective value of FBXL10, Ad‐FBXL10 was transfected into these cells for 24 hours, and subsequently normal (5.5 mmol/L) or high glucose (33 mmol/L) treatment was initiated. To inhibit PKC β2, the cells were treated with 1 μmol/L dose of PKC β2 kinase inhibitor.

### 2.5 Western blotting

In brief, the samples were lysed using RIPA buffer after two washes with cold PBS. The cell samples were loaded onto SDS‐PAGE gels, followed by transfer onto PVDF membranes (Amersham Bioscience, USA). After overnight incubation of the membranes with the specific primary antibodies at 4°C, 5% BSA was used for membrane blocking, and then the membranes were incubated with HRP‐conjugated secondary antibodies. An Enhanced Chemiluminescence Kit (Amersham Bioscience, USA) was then used to identify the reactive bands. The antibodies used were list as followed: FBXL10, Bcl‐2, Bax, p‐PKC β2 (Abcam), cleaved caspase 3, cleaved caspase 9, PKC β2, p67phox, and β‐actin (Cell Signaling Technology).

### 2.6 Reverse‐transcription and real‐time PCR

TRIzol was used for RNA isolation (Invitrogen), and RNA purity/quantity was determined by spectroscopy. For real‐time PCR, RNA was used to make cDNA with SuperScript II reverse transcriptase (Invitrogen). The SsoFasr^TM^ Probes Supermix (Bio‐Rad) was used to perform triplicate 20 μL PCR reactions using specific primer/probe sets as well as a 35 cycle protocol with a Bio‐Rad CFX96^TM^ Real‐time PCR Machine. The standard 2^−ΔΔCt^ method was used to assess changes in expression. The primers are list as followed: ICAM‐1: forward, 5′‐CAATTTCTCATGCCGCACAG‐3′, reverse, 5′‐AGCTGGAAGATCGAAAGTCCG‐3′; iNOS: forward, 5′‐GCAGAATGTGACCATCATGG‐3′, reverse, 5′‐ACAACCTTGGTGTTGAAGGC‐3′; p67phox, forward, 5′‐TTCCATCCCCAAATGCAAAG‐3′, reverse, 5′‐TCAGATGCCCTAAAACCGGAG‐3′; Gp91phox, forward, 5′‐GACCATT GCAAGTGAACACCC‐3′, reverse, 5′‐AAATGAAGTGGACTCCACGCG‐3′; β‐actin, forward, 5′‐GATGGCCACGGCTGCTTC‐3′, reverse, 5′‐TGCCTCAGGGCAGCGGAA‐3′.

### 2.7 Detection of oxidative activity

Rat hearts weighing 80‐120 mg were homogenized and spun down (4230 g, 10 minutes), and the supernatant was collected. Superoxide dismutase (SOD) activity, cAMP, NADPH oxidase activity, and lipid peroxidation levels were all measured using commercially available kits from Abcam.

### 2.8 ROS detection

For detection of ROS, the indicated cells were incubated with 2′,7′‐dichlorodihydrofluorescein diacetate (DCFH‐DA) for 30 minutes at 37°C. To quantify the ROS production, the fluorescence intensity was measured by flow cytometry.

### 2.9 Elisa

TNF‐α, IL‐1β and IL‐6 levels in the cell culture media were assessed via ELISA kits purchased from Abcam. The samples were analyzed in duplicate at room temperature.

### 2.10 Statistical analyses

All statistics are shown as the means ± SD. The One‐way ANOVA was used with a post hoc Tukey('s test for multiple group comparisons. When comparing two groups, two‐sided unpaired Student')s *t* tests were used. No samples were excluded from the analysis. The threshold for statistical significance was *P* < 0.05.

## 3 RESULTS

### 3.1 FBXL10 is downregulated in the cardiac tissue of STZ‐treated animals

**Figure 1:**
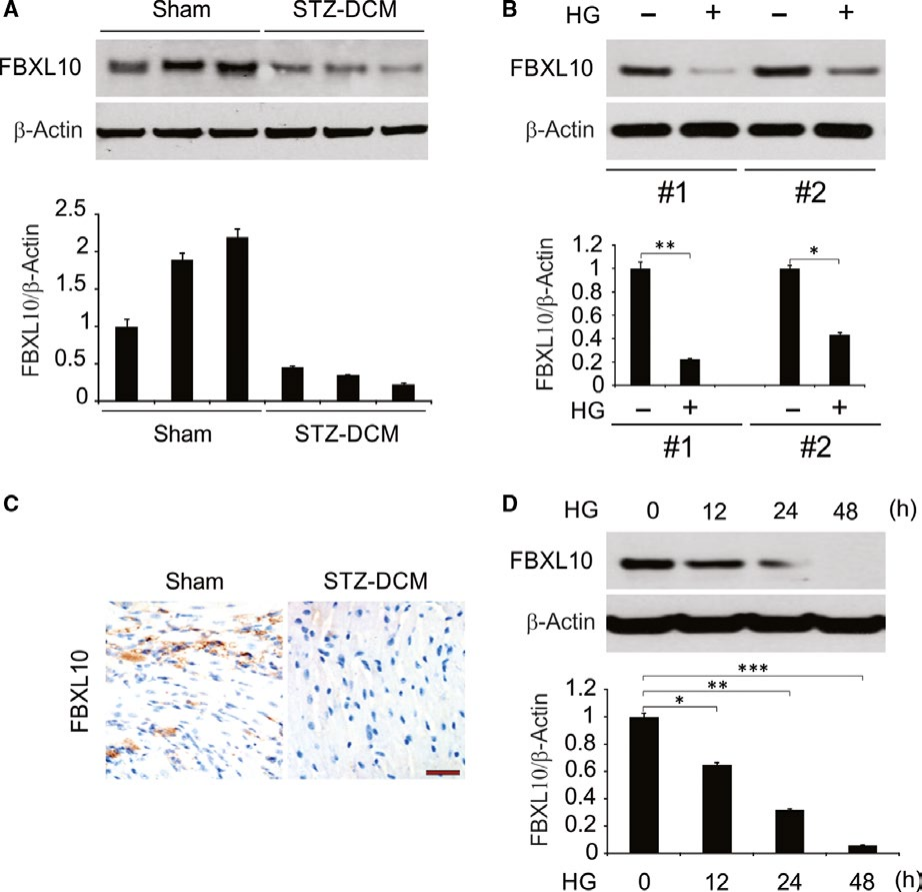
FBXL10 was downregulated in the heart in STZ‐induced diabetic mice, in HG‐stimulated cardiomyocytes and H9c2 cells. (A) The expression of FBXL10 in STZ‐induced diabetic hearts were analyzed by Western blotting and normalized to β‐actin. Data represent the mean ± SD of three independent experiments. (B) The protein level of FBXL10 in cardiomyocytes was analyzed by Western blotting and normalized to β‐actin. Data represent the mean ± SD of three independent experiments. **, *P *< 0.01; *, *P *< 0.05. (C) Immunohistochemistry of FBXL10 in STZ‐induced diabetic hearts. Scale bar, 50 μm (n = 6). (D) H9c2 cells were treated with high glucose (HG, 33 mmol/L glucose), FBXL10 level was analyzed by Western blotting and normalized to β‐actin. Data represent the mean ± SD of three independent experiments. **P < 0.01; *P < 0.05

FBXL10 expression levels were measured in diabetic rats treated with STZ to induce cardiac injury, and protein level of FBXL10 was found to be downregulated in this context (Figure 1A). FBXL10 protein levels were similarly lower in cardiomyocytes treated with HG to cause damage relative to control cells (Figure 1B). Cardiac FBXL10 levels were also reduced upon STZ injury in diabetic rats as measured by immunohistochemistry (Figure 1C). This finding suggests that FBXL10 may be linked in some way with diabetic cardiomyopathy. H9c2 cells were also stimulated with HG, and as shown in Figure 1D, compared with the control, HG could decrease the protein level of FBXL10 in a time‐dependent manner.

### 3.2 FBXL10 reduced HG‐associated inflammation and cell death in vitro

**Figure 2:**
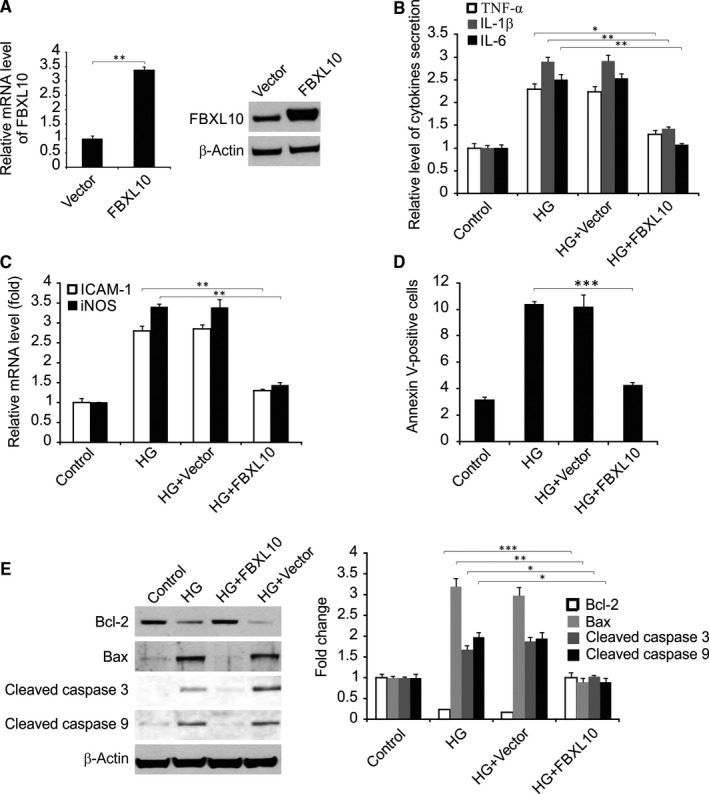
FBXL10 reduced HG‐induced inflammatory response and apoptosis. (A) H9c2 cells were transfected with FBXL10, FBXL10 expression was analyzed by Western blotting and real‐time RT‐PCR. Data represent the mean ± SD of three independent experiments. **P < 0.01. (B) H9c2 cells with or without FBXL10 transfection were stimulated by HG. The secretion of TNF‐α, IL‐1β and IL‐6 was determined by ELISA. Data represent the mean ± SD of three independent experiments. **P < 0.01; *P < 0.05. (C) H9c2 cells with or without FBXL10 transfection were stimulated by HG. The expression of ICAM‐1 and iNOS was assessed by real time RT‐PCR. Data represent the mean ± SD of three independent experiments. **P < 0.01. (D) H9c2 cells with or without FBXL10 transfection were stimulated by HG. Cell apoptosis was assessed by flow cytometry. Data represent the mean ± SD of three independent experiments. ***P < 0.001. (E) H9c2 cells with or without FBXL10 transfection were stimulated by HG. Indicated protein level was detected by Western blotting and normalized to β‐actin. Data represent the mean ± SD of three independent experiments. ***P < 0.001; **P < 0.01; *P < 0.05

To assess how FBXL10 functions in the context of HG‐induced inflammation, H9c2 cardiac cells were transfected to overexpress FBXL10 for 24 hours, after which, these cells were stimulated with HG for 48 hours. FBXL10 overexpression was confirmed by both real‐time RT‐PCR and Western blotting (Figure 2A). The HG treatment of H9c2 cells was associated with significant TNF‐α, IL‐1β and IL‐6 secretion as measured by ELISA, and FBXL10 overexpression significantly reduced inflammatory cytokine production (Figure 2B). FBXL10 overexpression was also linked to a reduction in the observed induction of mRNA level of iNOS and ICAM‐1 upon HG treatment of these cells (Figure 2C). These results thus suggest that FBXL10 is capable of reducing the inflammation associated with the HG stimulation of cardiac cells in vitro.

Flow cytometry analysis demonstrated that HG treatment was associated with a marked increase in cardiac cell apoptosis, but FBXL10 overexpression significantly attenuated this source of cell death (Figure 2D). HG treatment was linked to increase the level of Bax, cleaved caspase 3, and caspase 9, and Bcl‐2 downregulation. In addition, FBXL10 overexpression was sufficient to normalize the levels of these proteins (Figure 2E).

### 3.3 FBXL10 activated PKC β2 and reduced HG‐induced inflammation and apoptosis in H9c2 cells

**Figure 3:**
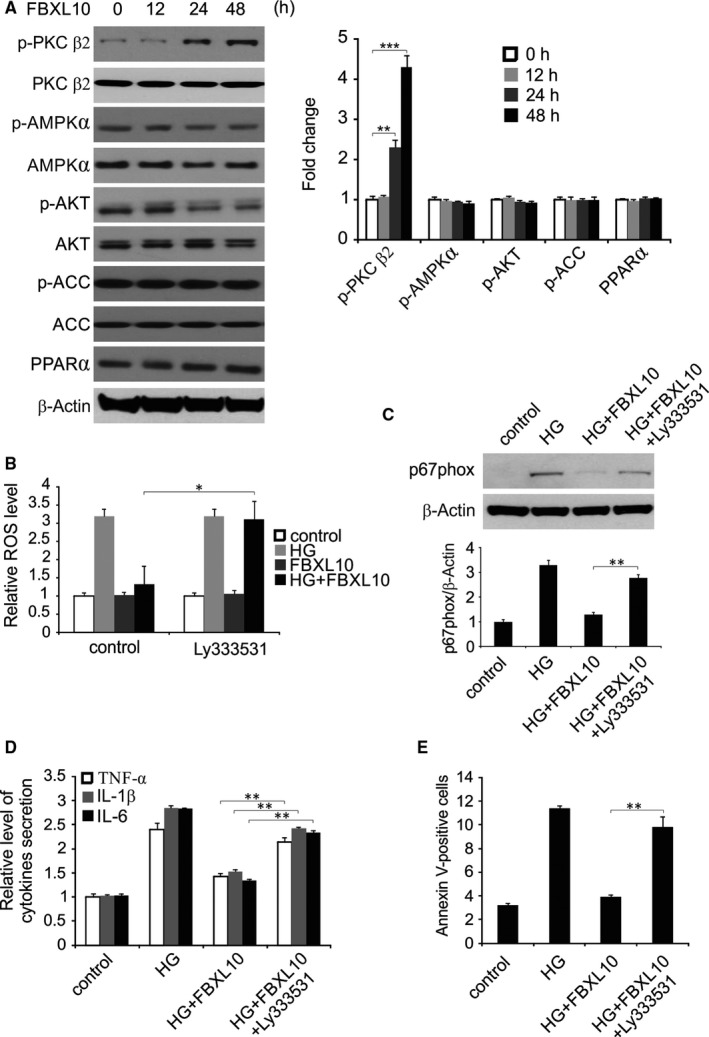
FBXL10 suppressed HG‐induced production of ROS, inflammation and apoptosis in H9c2 cells via PKC β2. (A) H9c2 cells were transfected with FBXL10. Indicated protein level was detected by Western blotting at indicated time point and normalized to β‐actin. Data represent the mean ± SD of three independent experiments. ***P < 0.001; **P < 0.01. (B) H9c2 cells transfected with FBXL10 were treated with HG with or without Ly333531. ROS was analyzed by DCFH‐DA. Data represent the mean ± SD of three independent experiments. *P < 0.05. (C) H9c2 cells transfected with FBXL10 were treated with HG with or without Ly333531. Protein level of P67phox were analyzed by Western blotting and normalized to β‐actin. Data represent the mean ± SD of three independent experiments. **P < 0.01. (D) H9c2 cells transfected with FBXL10 were treated with HG with or without Ly333531. The secretion of TNF‐α, IL‐1β and IL‐6 was determined by ELISA. Data represent the mean ± SD of three independent experiments. **P < 0.01. (E) H9c2 cells transfected with FBXL10 were treated with HG with or without Ly333531. Apoptosis was analyzed by Flow cytometry. Data represent the mean ± SD of three independent experiments. **P < 0.01

We next investigated the signaling pathway involved in the function of FBXL10 in vitro and found that FBXL10 overexpression significantly increased the phosphorylation of PKC β2. However, FBXL10 overexpression did not affect other signaling pathway investigated (Figure 3A). To determine whether increased PKC β2 activation was linked to the observed FBXL10‐associated cardiac cell protection, H9c2 cells were treated with the PKC β2 inhibitor LY333531, which protects against ROS production induced in these cells by FBXL10 (Figure 3B). The p67phox measurement supported the finding that FBXL10 attenuated the ROS production associated with HG treatment via PKC β2 activation (Figure 3C). PKC β2 deficiency similarly blunted the observed FBXL10‐dependent reductions in TNF‐α, IL‐1β and IL‐6 production by H9c2 cells upon HG treatment, suggesting that PKC β2 is linked with this inflammatory process (Figure 3D).

When H9c2 cell apoptosis was measured after HG treatment, we found that PKC β2 inhibition also abolished FBXL10‐mediated protection against HG‐induced apoptosis (Figure 3E).

### 3.4 FBXL10 attenuated STZ‐induced cardiac injury and improved cardiac function

**Figure 4:**
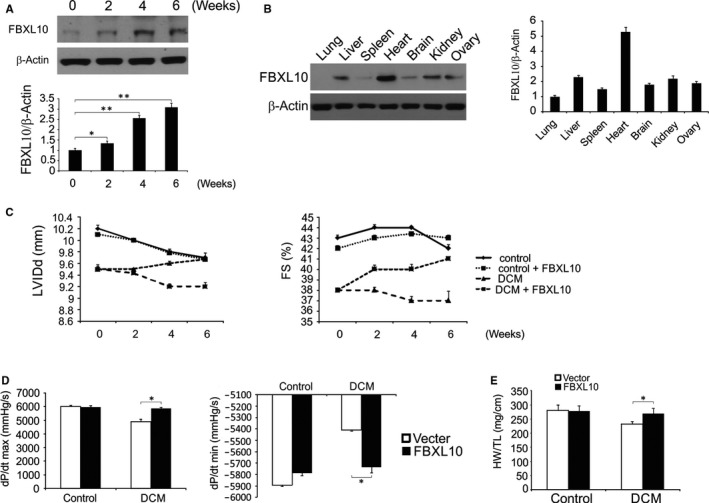
FBXL10 improved diabetes‐induced cardiac dysfunction in vivo. (A) FBXL10 overexpression in rat hearts after infection with an AAV was analyzed by Western blotting and normalized to β‐actin. Data represent the mean ± SD of three independent experiments. **P < 0.01; *P < 0.05. (B) The expression of FBXL10 in heart and extracardiac organs after transfection was analyzed by Western blotting and normalized to β‐actin. (C) Alteration of LVIDd and FS after overexpression of FBXL10 (n = 6). (D) Effect of on haemodynamic measurements (n = 6). (E) The ratio of heart weight (HW) to tibia length (TL) (n = 6). *P < 0.05

STZ‐treated diabetic rats presented with typical symptoms of diabetes^25^, ^28^; however, cardiac FBXL10 overexpression (Figure 4A) did not alter these symptoms (Table 1). Consistence with previously study,^29^ we also found FBXL10 expressed in other tissues. However the level is lower than heart (Figure 4B). At 12 weeks after STZ injection, and the rats presented with markedly decreased cardiac function with a significantly decrease in fractional shortening (FS) and left ventricular internal diastolic diameter (LVIDd). However, STZ‐induced cardiac dunction decrement could be improved by cardiac FBXL10 overexpression (Figure 4C). Relative to controls, STZ‐treated rats presented with both systolic dysfunction (dP/dt max) and diastolic dysfunction (dP/dt min) (Figure 4D), and FBXL10 overexpression attenuated these functional defects. Similarly, diabetic rats presented with a reduced heart to body weight ratio that was also partially rescued by FBXL10 overexpression (Figure 4E). The above findings demonstrate that FBXL10 ovexpression attenuated STZ‐induced cardiac dysfunction.

**Table 1:** The role of FBXL10 on blood glucose and body weight in DCM rats

	Control	Control + FBXL10	DCM	DCM + FBXL10
Blood glucose (mmol/L)				
Before FBXL10 overexpression	4.56 (0.31)	4.44 (0.32)	25.09 (1.56)	25.02 (1.49)
2 Weeks after FBXL10 overexpression	5.01 (0.38)	4.98 (0.37)	25.22 (1.71)	25.17 (1.59)
Body weight (g)				
Before FBXL10 overexpression	559.7 (23.2)	560.3 (23.9)	422.1 (17.2)	423.4 (16.9)
2 Weeks after FBXL10 overexpression	612.3 (24.9)	610.7 (25.5)	427.2 (19.2)	429.4 (20.4)

Data were shown as mean (SD).

### 3.5 Overexpression of FBXL10 in diabetic cardiac tissue reduced oxidative damage and inflammation

**Figure 5:**
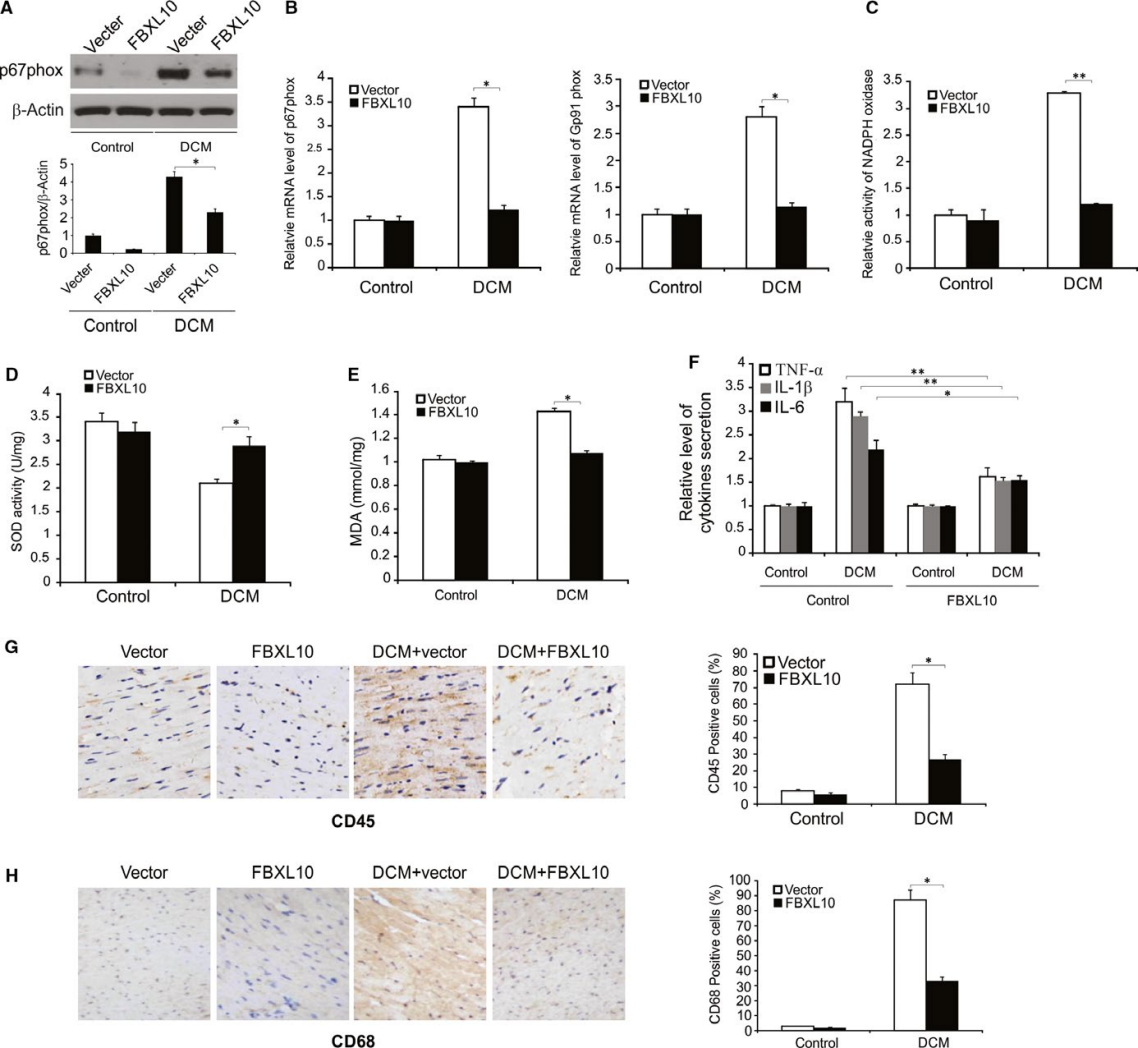
FBXL10 attenuated diabetes‐induced oxidative injury and inflammation in vivo. (A) The expression of p67phox in diabetic rat hearts was analyzed by Western blotting and normalized to β‐actin. Data represent the mean ± SD of three independent experiments. *P < 0.05. (B) The mRNA level of P67phox and Gp91phox in diabetic hearts was analyzed by Real‐time PCR. Data represent the mean ± SD of three independent experiments. *P < 0.05. (C) NADPH oxidase activity in diabetic hearts by FBXL10 overexpression. Data represent the mean ± SD of three independent experiments. **P < 0.01. (D) Total SOD activity in diabetic hearts after FBXL10 overexpression. Data represent the mean ± SD of three independent experiments. *P < 0.05. (E) Lipid peroxidation in diabetic hearts. Data represent the mean ± SD of three independent experiments. *P < 0.05. (F) mRNA level of myocardial TNF‐α, IL‐1β and IL‐6 in rats with diabetes. Data represent the mean ± SD of three independent experiments. **P < 0.01; *P < 0.05. (G) CD45 expression in the rat heart with diabetes was analyzed by immunohistochemistry. Data represent the mean ± SD of three independent experiments. *P < 0.05. (H) CD68 expression in the rat heart with diabetes was analyzed by immunohistochemistry. Data represent the mean ± SD of three independent experiments. *P < 0.05

FBXL10 overexpression was linked to significant reductions in NADPH oxidase component p67 expression as assessed by Western blotting in rat cardiac tissue (Figure 5A). Similarly, at the mRNA level STZ‐associated p67phox and Gp91phox upregulation was reduced by FBXL10 overexpression (Figure 5B). FBXL10 overexpression in the diabetic rats also reduced the abnormal NADPH oxidase activity (Figure 5C). Relative to control rats, total cardiac SOD activity was significantly reduced in diabetic rats, and FBXL10 overexpression increased this activity (Figure 5D). FBXL10 overexpression also reduced myocardial lipid peroxidation in these diabetic rats (Figure 5E). FBXL10 also significantly altered myocardial TNF‐α, Il‐1β and Il‐6 levels in these rats (Figure 5F). In addition, the increased CD68‐labeled macrophage and CD45‐labeled leukocyte infiltration was reduced by FBXL10 overexpression in STZ‐induced diabetic rat hearts (Figure 5G,H). These findings demonstrate that FBXL10 acts in the cardiac tissue of diabetic rats to reduce oxidative tissue damage and inflammation.

### 3.6 FBXL10 reduced diabetes‐associated cardiac cell death

**Figure 6:**
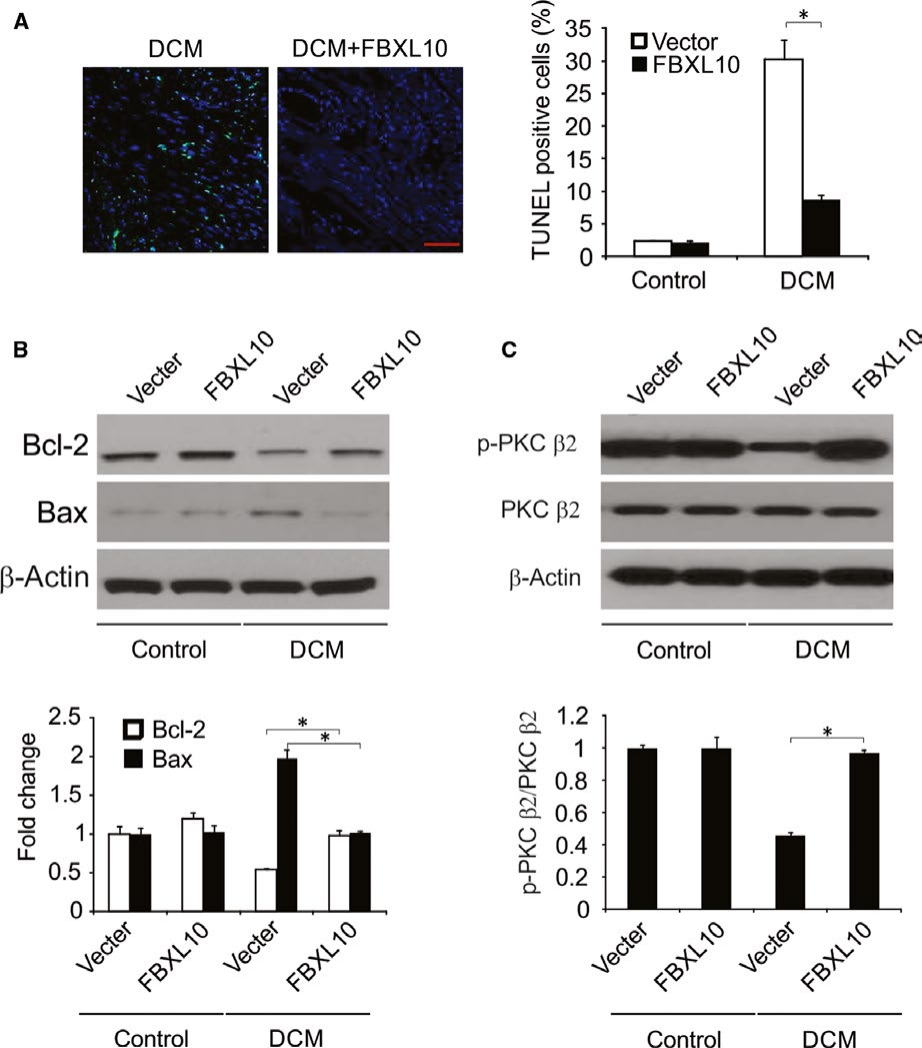
FBXL10 attenuated diabetes‐induced apoptosis in vivo. (A) TUNEL staining in diabetic hearts (n = 6). Scale bar, 20 μm. (B) The expression of Bcl‐2 and Bax in diabetic hearts was analyzed by Western blotting and normalized to β‐actin. Data represent the mean ± SD of three independent experiments. *P < 0.05. (C) The expression of phosphor‐ and totle‐PKC β2 in the diabetic hearts was analyzed by Western blotting. The level of p‐PKC β2 was normalized to PKC β2. Data represent the mean ± SD of three independent experiments. *P < 0.05

A significant number of apoptotic cells were present in the cardiac tissue of STZ‐treated rats, and FBXL10 reduced this cell death frequency (Figure 6A). Western blotting further confirmed that FBXL10 overexpression was associated with Bcl‐2 upregulation and Bax downregulation (Figure 6B).

### 3.7 FBXL10 activated PKC in diabetic hearts

We next investigated whether FBXL10 activated PKC in vivo. We found that diabetic rats had significantly reduced cardiac PKC β2 phosphorylation relative to that in controls, and FBXL10 was able to significantly increase this phosphorylation (Figure 6C).

## 4 DISCUSSION

Diabetic cardiomyopathy (DCM) is associated with a range of structural, cellular, and molecular changes that all act to mediate cardiac tissue dysfunction,^30^, ^31^ but the precise molecular mechanisms governing this serious disease remain to be fully elucidated. To our knowledge, our study is the first report to indicate that overexpression of FBXL10 in the heart protects against diabetes‐related cardiac dysfunction, cell death, inflammation and oxidative damage. In particular, we found that treatment with high glucose (HG) was associated with elevated oxidative stress and decreased FBXL10 expression in cardiac cells. In addition, FBXL10 overexpression was sufficient to reduce the inflammation associated with HG treatment, decreasing the production of the inflammatory cytokines TNF‐α, IL‐1β and IL‐6 and normalizing the expression of ICAM‐1 and iNOS. We also found FBXL10 overexpression was also associated with reduced cardiomyocyte apoptosis in response to HG treatment, a reduction attributed to reduce cleaved caspase 3 and caspase 9 levels, and altered PKC β2 pathway activation. Finally, FBXL10 overexpression in vivo was sufficient to restore cardiac function, reducing the cell death and inflammatory response associated with hyperglycemia in these animals.

Previously study has reported the relationship between FBXL10 and development including heart defects.^32^ Some studies also found that the expression level of FBXL10 association cell death regulation.^33^, ^34^ Importantly, we found that STZ‐treated diabetic rodents presented with both impaired diastolic and systolic cardiac functions, and FBXL10 overexpression improved these functions and reduced diabetes‐associated cardiac injury accordingly. Our data thus suggest that FBXL10 may serve a physiological role in protecting cardiac tissue from DCM in vivo.

Sharp increases in oxidative stress are thought to be among the key drivers of DCM progression, with elevated ROS production in myocytes in response to HG leading to increased DNA damage, lipid peroxidation, cell death, and consequent cardiac dysfunction.^35^, ^36^ Identifying factors that can suppress oxidative stress is thus invaluable.^37^ We have provided clear evidence that FBXL10 can protect against HG‐induced ROS production in vitro. Similarly, cardiac tissue in diabetic animals exhibits increased inflammation, with higher levels of TNF‐α, IL‐6 and IL‐1β, all of which can profoundly alter cardiac function.^9^, ^38^, ^39^ We found that FBXL10 overexpression protected cardiac tissue against inflammation, underscoring the protective role of FBXL10 in the context of cell death associated with DCM.

Cell death is a key factor of STZ‐induced cardiomyopathy.^39^, ^40^ Previously study reported that FBXL10 regulates cell death as an anti‐apoptotic protein and suppress TRAIL‐induced apoptosis.^41^ In the current study, we found overexpression of FBXL10 inhibited HG‐induced apoptosis in H9c2 cells and STZ‐induced apoptosis in animal model. Overall, our results indicate that the protective role of FBXL10 against cell death in diabetic hearts.

PKC activation in cardiomyocytes drives the expression of numerous distinct genes owing to the activation of many key signal transduction pathways.^42^, ^43^ PKC overexpression or overactivation have previously been linked to the induction of expression of proto‐oncogenes and transcription factors in cardiac tissue, ultimately facilitating cardiac damage and interstitial fibrosis via altered collagen production and tissue regulation.^44^, ^45^ PKC‐α and PKC‐β2 overexpression has been identified in individuals presenting with pathological DCM, and such overexpression might to be linked to hyperglycemia‐mediated activation of the DAG‐PKC signal transduction pathway, elevating cardiomyocyte PKC expression and altering PKC distribution within cells to promote the association of this protein with the cell membrane.^24^ We found that FBXL10 activated PKC β2 in vivo and in vitro, and we hypothesize that the abovementioned protective effects of FBXL10 are mediated by PKC β2. Notably, PKC β2 inhibition abolished the protective effects of FBXL10 against oxidative stress, inflammation and cell death.

In conclusion, this study serves to clarify the regulatory role of FBXL10 in diabetic heart inflammation, oxidative stress and apoptosis, demonstrating that FBXL10 could be a promising therapeutic approach for the treatment of DCM.

## CONFLICT OF INTEREST

The authors confirm that there are no conflicts of interest.
